# Phase 1 trial of hypofractionated stereotactic re-irradiation in combination with nivolumab, ipilimumab, and bevacizumab for recurrent high-grade gliomas

**DOI:** 10.1093/noajnl/vdaf033

**Published:** 2025-02-08

**Authors:** Solmaz Sahebjam, Raju R Raval, Peter A Forsyth, Heiko Enderling, Nam D Tran, John A Arrington, Robert Macaulay, Haley K Perlow, Joshua D Palmer, Jayeeta Ghose, Prajwal Rajappa, Pierre Giglio, Zihai Li, Arnold B Etame, Sepideh Mokhtari, Ruben J Cruz-Chamorro, Menal Bhandari, Ram Thapa, Timothy J Robinson, Dung-Tsa Chen, Hsiang-Hsuan Michael Yu

**Affiliations:** Johns Hopkins University School of Medicine, The Sidney Kimmel Cancer Center, Sibley Memorial Hospital, Washington, DC, USA; H. Lee Moffitt Cancer Center and Research Institute, Tampa, Florida, USA; Pelotonia Institute for Immuno-Oncology, Columbus, Ohio, USA; The Ohio State University Wexner Medical Center, James Cancer Hospital, Columbus, OH, USA; H. Lee Moffitt Cancer Center and Research Institute, Tampa, Florida, USA; The University of Texas MD Anderson Cancer Center, Houston, Texas, USA; H. Lee Moffitt Cancer Center and Research Institute, Tampa, Florida, USA; H. Lee Moffitt Cancer Center and Research Institute, Tampa, Florida, USA; H. Lee Moffitt Cancer Center and Research Institute, Tampa, Florida, USA; Department of Radiation Oncology, University Hospitals Seidman Cancer Center, Case Western Reserve University, Cleveland, Ohio, USA; The Ohio State University Wexner Medical Center, James Cancer Hospital, Columbus, OH, USA; The Ohio State University Wexner Medical Center, James Cancer Hospital, Columbus, OH, USA; The Ohio State University Wexner Medical Center, James Cancer Hospital, Columbus, OH, USA; Pelotonia Institute for Immuno-Oncology, Columbus, Ohio, USA; The Ohio State University Wexner Medical Center, James Cancer Hospital, Columbus, OH, USA; The Ohio State University Wexner Medical Center, James Cancer Hospital, Columbus, OH, USA; Pelotonia Institute for Immuno-Oncology, Columbus, Ohio, USA; The Ohio State University Wexner Medical Center, James Cancer Hospital, Columbus, OH, USA; H. Lee Moffitt Cancer Center and Research Institute, Tampa, Florida, USA; H. Lee Moffitt Cancer Center and Research Institute, Tampa, Florida, USA; H. Lee Moffitt Cancer Center and Research Institute, Tampa, Florida, USA; H. Lee Moffitt Cancer Center and Research Institute, Tampa, Florida, USA; H. Lee Moffitt Cancer Center and Research Institute, Tampa, Florida, USA; Yale School of Medicine, Smilow Cancer Center, New Haven, Connecticut, USA; H. Lee Moffitt Cancer Center and Research Institute, Tampa, Florida, USA; H. Lee Moffitt Cancer Center and Research Institute, Tampa, Florida, USA

**Keywords:** glioblastoma, high-grade glioma, ipilimumab, nivolumab, stereotactic re-irradiation

## Abstract

**Background:**

Our previous clinical investigation suggested that hypofractionated stereotactic re-irradiation (HFSRT) and PD-1 blockade may act synergistically to enhance the immune response against glioma. This subsequent trial investigated the dual blockade of CTLA4 and PD-1 in combination with HFSRT and bevacizumab.

**Methods:**

This phase I study enrolled eligible patients with bevacizumab-naïve recurrent glioblastoma or anaplastic astrocytoma. Participants received nivolumab, ipilimumab, and bevacizumab concurrently with HFSRT (3000 cGy in 5 fractions). Subsequently, nivolumab, ipilimumab, and bevacizumab were administered for a total of 4 cycles followed by nivolumab and bevacizumab until progression. The primary end point of this study was the safety and tolerability of HFSRT in combination with nivolumab, ipilimumab, and bevacizumab in patients with recurrent HGGs. Secondary end points included 6-month survival and 9-month survival.

**Results:**

Twenty-six patients were treated. Treatment-related adverse events (TRAEs) of grade 3 or 4 were observed in 12 (48%) evaluable patients with no unexpected TRAEs. Six months and 9 months survival were 92% (95% CI, 82–100%) and 75% (95% CI, 60–95%), respectively. The median progression-free survival and overall survival were 7.1 months (95% CI, 5.2–12.2) and 15.6 months (95% CI, 11.3–27.0), respectively.

**Conclusions:**

The combination of HFSRT with ipilimumab, nivolumab, and bevacizumab is safe. Our results underscore the potential synergies between stereotactic re-irradiation and checkpoint immunotherapy in patients with recurrent high-grade gliomas.

**Key Points**

- Hypofractionated stereotactic re-irradiation in combination with nivolumab, ipilimumab, and bevacizumab in patients with recurrent bevacizumab-naïve high-grade glioma is safe.
- The median overall survival was 15.6 months (95% CI, 11.3–27.0) months.

**Importance of the Study**

Preclinical and clinical investigations have suggested that radiotherapy and immunotherapy may act synergistically to enhance the immune response against cancer cells. Our previous clinical trial demonstrated the safety and efficacy of combining stereotactic radiotherapy with an anti-PD-1 antibody and an anti-angiogenic agent in patients with recurrent high-grade glioma. Furthermore, in murine glioblastoma models, administration of anti-CTLA-4 plus anti-PD-1 mAb therapy resulted in markedly superior long-term survival when compared with monotherapy of either agent alone. In this clinical trial, we combined hypofractionated stereotactic radiotherapy with an anti-CTLA4 antibody (ipilimumab), an anti-PD-1 antibody (nivolumab), and an anti-angiogenic agent (bevacizumab) in patients with bevacizumab-naïve recurrent high-grade glioma. Median OS was 15.6 months and ~62% of subjects were alive 12 months after starting study treatment. This study, combined with our previously published trial, underscores the potential benefit of utilizing hypofractionated stereotactic radiation in potentiating the efficacy of immunotherapy for patients with recurrent high-grade gliomas.

High-grade gliomas (HGGs) including glioblastoma (GBM), account for the majority of malignant primary central nervous system (CNS) tumors in adults.^1^ Despite many decades of focused translational research and numerous clinical trials, the prognosis for patients with HGGs remains poor, with a 5-year relative survival rate of 6.9% for GBM patients.^1^ Few treatment options are available when HGG recurs. Novel treatment strategies are urgently needed for patients with newly diagnosed and recurrent HGG.

The tumor microenvironment in patients with GBM is known to be immunosuppressive. Previous preclinical investigations with glioma models have shown strong anti-tumor activity of anti-PD-1/anti-PD-L1 blockade alone or in combination with anti-CTLA4 antibodies.^2,3^ However, several phase II and phase III clinical trials failed to show a survival benefit for single agent anti-PD-1/PD-L1 therapy in patients with recurrent GBM.^4,5^ Median overall survival (OS) for patients with first recurrence of GBM who were treated with anti-PD-1/PD-L1 inhibitors was approximately 9 months, which was not significantly different from the standard of care controls.^4^ These disappointing clinical results highlight the need for finding strategies that can enhance the immunostimulatory effects of immune checkpoint inhibitors in the setting of GBM and other HGGs.

Preclinical and clinical investigations have suggested that radiotherapy and immunotherapy may act synergistically to enhance the immune response against cancer cells.^3,6–9^ The combination of radiation therapy with PD-1/ PD-L1 blockade can result in the activation of cytotoxic T-cells, reduction of myeloid-derived suppressor cells, and enhanced treatment response both in and outside the radiation field.^10–12^ Higher doses per fraction have also been associated with higher tumor-specific T cell response, lower regulatory T cells, increased ratio of cytotoxic T cells to regulatory T cells, and improved local tumor control.^3,13,14^ However, the optimal timing and the dose/fractionation of radiotherapy when combining with immunotherapy in humans is not yet fully determined. In a phase 1 clinical trial (ClinicalTrials.gov identifier: NCT02313272) that we previously published, we demonstrated the safety and efficacy of combining hypofractionated stereotactic radiotherapy (HFSRT) of 3000 cGy in 5 fractions with an anti-PD-1 antibody (pembrolizumab) and an anti-angiogenic agent (bevacizumab) in patients with recurrent HGGs including GBMs.^9^ In that trial, we employed a radiation regimen of 600 cGy × 5 fractions that is considered as a moderate hypofractionated regimen thought to be immunogenic and synergistic with checkpoint inhibitors. Exploratory analysis of anti-tumor efficacy was encouraging with median progression-free survival (PFS) of 7.92 months (95% CI: 6.31–12.45) and a median OS of 13.45 months (95% CI: 9.46–18.46) in patients with bevacizumab-naïve recurrent HGGs.^9^

Here, we present the safety and efficacy results of a phase 1, multicenter clinical trial investigating HFSRT in combination with nivolumab, ipilimumab, and bevacizumab for patients with recurrent HGGs (ClinicalTrials.gov identifier: NCT02829931). This combination was based on findings from preclinical studies in murine glioblastoma models in which administration of anti-CTLA-4 plus anti-PD-1 mAb therapy resulted in markedly superior long-term survival and median OS when compared with monotherapy of either agent alone.^2^ Moreover, clinical trials from other malignancies such as melanoma and lung cancer have demonstrated significant anti-tumor and OS benefit following dual blockade of CTLA4 and PD-1.^15,16^ Bevacizumab, an anti-vascular endothelial growth factor (VEGF) antibody was added for enhancing the benefit from immunotherapy based on accumulating evidence underlining the role of VEGF as a mediator of tumor-induced immunosuppression.^17–20^ Furthermore, the addition of bevacizumab was aimed to mitigate potential side effects of re-irradiation and prevent the need for corticosteroids, in the setting of potential treatment-induced edema following re-irradiation, which can have negative impacts on the therapeutic effect of checkpoint inhibitors in primary and metastatic brain malignancies.^4,21^

## Patients and Methods

This single-arm, open-label phase 1 trial was conducted at the Moffitt Cancer Center and The Ohio State University Wexner Medical Center between 2016 and 2021 and was compliant with the Declaration of Helsinki and guidelines on Good Clinical Practice. The protocol and its amendments were reviewed and approved by the institutional review boards and ethics committees. All patients provided written informed consent. This study was discontinued in 2021 as per the sponsor’s decision.

### Patients

Patients were eligible if they were ≥ 18 years of age and had a recurrent World Health Organization (WHO) grade 3 or grade 4 glioma; maximum diameter of gadolinium-enhancing tumor (target lesion) ≤ 4 cm; previous first-line treatment with at least standard dose of radiotherapy (total dose ≥ 54 Gy) and temozolomide or PCV (Procarbazine, Lomustine, and Vincristine) chemotherapy; an interval of at least 6 months after the end of prior radiation therapy unless there was a new recurrence outside of the previous radiotherapy treatment field; Karnofsky performance status (KPS) score of 70 or above; and adequate pulmonary, liver, kidney, and bone marrow function. Prior treatment with PCV was allowed since patients with recurrent grade 3 oligodendroglioma were considered eligible. Patients were excluded if they had more than 3 recurrences of HGG; received re-irradiation to recurrent disease (in addition to standard frontline definitive radiation therapy); had prior treatment with bevacizumab or other anti-angiogenic agents; had tumor recurrence within 5 mm of the brainstem and/or the optic chiasm which had received prior radiation therapy; had evidence of infra-tentorial or leptomeningeal disease; active, known, or suspected autoimmune disease; had a history of gastrointestinal bleeding or any other hemorrhage/bleeding adverse event of grade ≥ 3 (Common Terminology Criteria for Adverse Events [CTCAE] v4) within 30 days prior to trial enrollment; had prior history of uncontrolled hypertension, hypertensive crisis or hypertensive encephalopathy; had history of non-healing wound, ulcer, or bone fracture within 90 days; or required chronic supraphysiologic doses of corticosteroids (> 10 mg/day prednisone equivalents) at the start day of treatment.

### Study Design and Treatment

**Figure 1.**
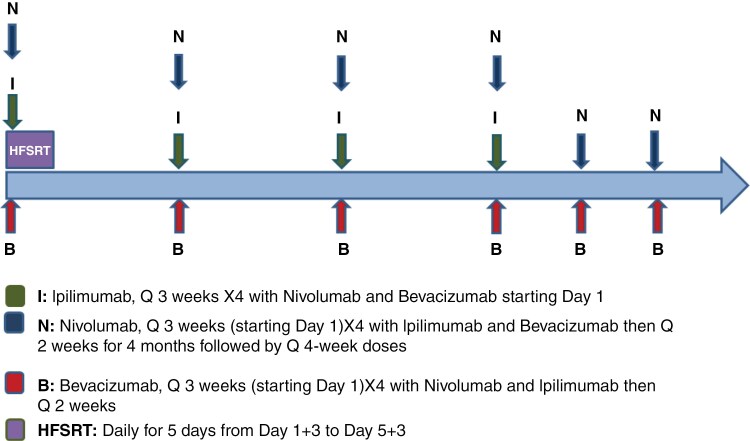
Study treatment.

This study was designed to investigate the safety and efficacy of HFSRT combined with nivolumab, ipilimumab, and bevacizumab (Figure 1). A safety lead-in enrolling 6 patients was performed to establish the safety of the trial treatment regimen prior to an expansion cohort. HFSRT started on C1D1 and was delivered once daily (M-F) in one week. The first doses of nivolumab (3 mg/kg i.v.), ipilimumab (1mg/kg i.v.), and bevacizumab (15 mg/kg i.v.) were administered on C1D1. Subsequently, nivolumab (3 mg/kg i.v.), ipilimumab (1mg/kg i.v.), and bevacizumab (15 mg/kg i.v.) were administered every 3 weeks for total of 4 cycles followed by nivolumab (240 mg i.v.) and bevacizumab (10 mg/kg i.v.) every 2 weeks for 4 months. After 4 months, nivolumab was administered every 4 weeks at 480 mg i.v. and bevacizumab was continued at every 2-week schedule. Patients continued ipilimumab (4 cycles), nivolumab, and bevacizumab until confirmed disease progression, intolerable toxicity, or withdrawal of consent. Dose reductions were not permitted. All three study treatments could be held for toxicity and restarted when toxicity resolved.

#### Radiation technique.

—CT simulation was performed by acquiring non-contrast CT of the head with 1.0–1.5 mm slice thickness after being immobilized with a non-invasive thermoplastic mask. Volumetric contrast-enhancing MRI of the brain with 1 mm slices was performed within 1 week of CT simulation for treatment planning and was fused with simulation CT images. Gross tumor volume (GTV) was defined as the enhancing tumor on T1 postcontrast imaging. For the patients who underwent repeat resection for recurrence immediately prior to enrollment of this study, the new resection cavity was also included in the GTV. The GTV was expanded by 3–5 mm margin to create the planning target volume (PTV). The PTV was prescribed to receive 3000 cGy in 5 daily fractions with at least 95% coverage. The protocol allowed that up to 0.5 cm^3^ volume of gross tumor to receive 4000 cGy in 5 fractions as a mean to enhance immune response using a simultaneous integrated boost technique. Treatment planning was performed with the Varian Eclipse, BrainLAB, or Pinnacle treatment planning systems. A single isocenter plan was used for each patient, and an intensity-modulated radiation therapy or volumetric arc radiation therapy technique was utilized for planning. The plan was normalized so that 100% of the prescription dose covered 95% of the PTV. Daily image guidance prior to each fraction was performed with either ExacTrac stereotactic x-ray system or cone-beam CT scans.

The reported rate for grade 3 CNS toxicities with HFSRT and bevacizumab is 4%.^22^ We considered treatment-related grade 3 CNS toxicity rate of > 10% excessively toxic and not suitable for further clinical development.

### End Points and Assessments

The primary end point was the safety and tolerability of HFSRT in combination with nivolumab, ipilimumab, and bevacizumab in patients with recurrent HGGs. Treatment-related adverse events (TRAEs) were graded according to Common Terminology Criteria for Adverse Events (CTCAE), version 4.0. All TRAEs were captured and recorded from the first day of immunotherapy treatment to 30 days following the last dose.

Secondary end points included objective response rate (ORR), 6 months survival and 9 months survival. All patients were evaluated for disease progression every 6 weeks (± 14 days) using MRI with and without contrast and according to Response Assessment in Neuro-Oncology (RANO) criteria.^23^ To decrease the likelihood of prematurely discontinuing potentially effective therapy, subjects with suspected radiologic disease progression were permitted to receive study treatment until disease progression was confirmed by repeat brain MRI performed approximately 8 weeks after the initial radiological assessment suggestive of progressive disease. If the follow-up MRI confirmed the progression of the disease, the date of initial determination was recorded as the date of tumor progression. Furthermore, treatment responses based on Immunotherapy RANO (iRANO) criteria were collected as exploratory data.^24^ For an exploratory analysis, all follow-up MRI imaging studies for patients treated at Moffitt Cancer Center were reviewed by radiation oncology physicians (RC and HMY) against treatment planning plans to determine the pattern of progression. Infield/marginal progression was defined as new T1-post-enhancing abnormality demonstrated within the 80% isodose lines during the follow-up imaging evaluation.

### Tumor Sample Analysis

Data on tumor O^6^-methylguanine DNA methyltransferase (*MGMT*) promoter methylation and isocitrate dehydrogenase (*IDH*) mutation status were collected in all patients. Molecular testing for *IDH1/2* mutations was performed either by polymerase chain reaction or pyrosequencing assay. Moreover, for patients with available tumor samples, tumor mutation burden (TMB) and microsatellite status were determined through commercial FoundationOne or FoundationOne CDx tumor testing when possible.

### Statistical Analysis

Safety and efficacy end points were assessed in all patients who completed HFSRT and received any dose of nivolumab, ipilimumab, or bevacizumab. Survival follow-ups were performed every 3 months. Objective response rate and disease control rate were summarized with 95% CI using Wilson score method. Progression-free survival (PFS) and overall survival (OS) were analyzed as ad hoc exploratory objectives. PFS was defined as the time from the date of treatment initiation to the date of documented progression or death, whichever occurred first. OS was defined as the time from the date of study treatment initiation to the date of death from any cause. PFS and OS were analyzed by Kaplan–Meier estimates and reported with 2-sided 95% CIs. The sample size of 26 patients was determined to ensure a 9-month overall survival rate of 80% with 95% CI of 60% to 93%.

## Results

### Patients

From August 2016 to December 2020, a total of 26 patients received study treatment. One patient started a new investigational treatment in another center weeks after the first study treatment administered on this trial. This patient was deemed unevaluable for toxicity or efficacy analyses. Treatments were administered at Moffitt Cancer Center and The Ohio State University Wexner Medical Center. Summary demographics and baseline disease characteristics are listed in Table 1.

**Table 1.** Patient Demographics and Clinical Characteristics

Characteristics	Patients (*N* = 25)
Age	
Median (range)	59 (27–77)
Sex, *n* (%)	
Male	19 (76)
Female	6 (24)
Histopathologic Diagnosis, *n* (%)	
Glioblastoma	22 (88)
Anaplastic Astrocytoma	3 (12)
KPS, *n* (%)	
100	1 (4)
90	8 (32)
80	10 (40)
70	3 (12)
Unknown	3 (12)
*MGMT* promoter methylation status, *n* (%)	
Methylated	8 (32)
Unmethylated	16 (64)
Unknown	1 (4)
*IDH* mutation status, *n* (%)	
Mutant	2 (8), both (IDH1 R132H)
Wildtype	23 (92)
Resection prior to study treatment, *n* (%)	
Yes	12 (48)
No	13 (52)
Recurrence(s), *n* (%)	
1^st^	18 (72)
2^nd^	6 (24)
3^rd^	1 (4)

Abbreviations: KPS, Karnofsky Performance Status scores; *MGMT*, *O*^6^-methylguanine DNA methyltransferase; IDH, isocitrate dehydrogenase.

At the time of analysis, all patients treated with HFSRT, nivolumab, ipilimumab, and bevacizumab had discontinued study therapy, including 64% (*n* = 16) due to progressive disease. Other reasons for discontinuation of study treatment included treatment-related toxicity (*n* = 4, 16%), patient preference or alternative therapy (*n* = 3, 12%), a medical condition unrelated to study treatment (*n* = 1, 4%), and study closure (*n* = 1, 4%). The median duration of time on study treatment and time on the study were 5.3 months (range, 0.7–17.4) and 11.8 months (range, 2.7–27.1), respectively.

### Safety

TRAEs of grade 3 or 4 were observed in 12 (48%) out of 25 evaluable patients. The most common TRAE events were hypertension and asymptomatic elevation of amylase (Table 2). The most common TRAEs of all grades were fatigue, diarrhea, hypertension, and elevation of alanine aminotransferase (Table 2). No unexpected toxicity was observed. Four patients discontinued study treatment due to TRAEs. Colitis and confusion were the TRAEs leading to treatment discontinuation. No case of radiographic pseudo-progression or symptomatic radiation necrosis was observed following re-irradiation.

**Table 2.** Treatment-Related Adverse Events

Treatment-Related Adverse Events	Any Grade *n* (%)	Grade 3/4 *n* (%)
Fatigue	17 (68)	3 (12)
Diarrhea	11 (44)	0
Hypertension	10 (40)	5 (20)
Alanine aminotransferase increased	9 (36)	1 (4)
Lipase increased	8 (32)	2 (8)
Proteinuria	8 (32)	2 (8)
Other	8 (32)	0
Aspartate aminotransferase increased	7 (28)	1 (4)
Serum amylase increased	7 (28)	3 (12)
Arthralgia	6 (24)	0
Pruritus	6 (24)	0
Rash	6 (24)	0
Hypothyroidism	5 (20)	0
Nausea	5 (20)	0
Alopecia	4 (16)	0
Abdominal pain	3 (12)	0
Anorexia	3 (12)	0
Colitis	3 (12)	2 (8)
Headache	3 (12)	0
Alkaline phosphatase increased	2 (8)	0
Chills	2 (8)	0
Confusion	2 (8)	1 (4)
Dysgeusia	2 (8)	0
Edema limbs	2 (8)	0
Fever	2 (8)	0
Flu like symptoms	2 (8)	0
Acute kidney injury	1 (4)	1 (4)
Adrenal insufficiency	1 (4)	0
Anemia	1 (4)	0
Blood and lymphatic system disorders—Other, specify	1 (4)	0
Blood bilirubin increased	1 (4)	0
Bruising	1 (4)	0
Dysphagia	1 (4)	1 (4)
Edema face	1 (4)	0
Epistaxis	1 (4)	0
Erythema multiforme	1 (4)	1 (4)
Generalized muscle weakness	1 (4)	0
Hematuria	1 (4)	0
Hyperthyroidism	1 (4)	0
Hypokalemia	1 (4)	1 (4)
Infusion related reaction	1 (4)	0
Joint range of motion decreased	1 (4)	0
Lymphocyte count decreased	1 (4)	0
Myalgia	1 (4)	0
Pain	1 (4)	0
Pain in extremity	1 (4)	0
Stroke	1 (4)	1 (4)
Vomiting	1 (4)	0
Weight loss	1 (4)	0
Total	**146**	**25**

### Efficacy

**Figure 2.**
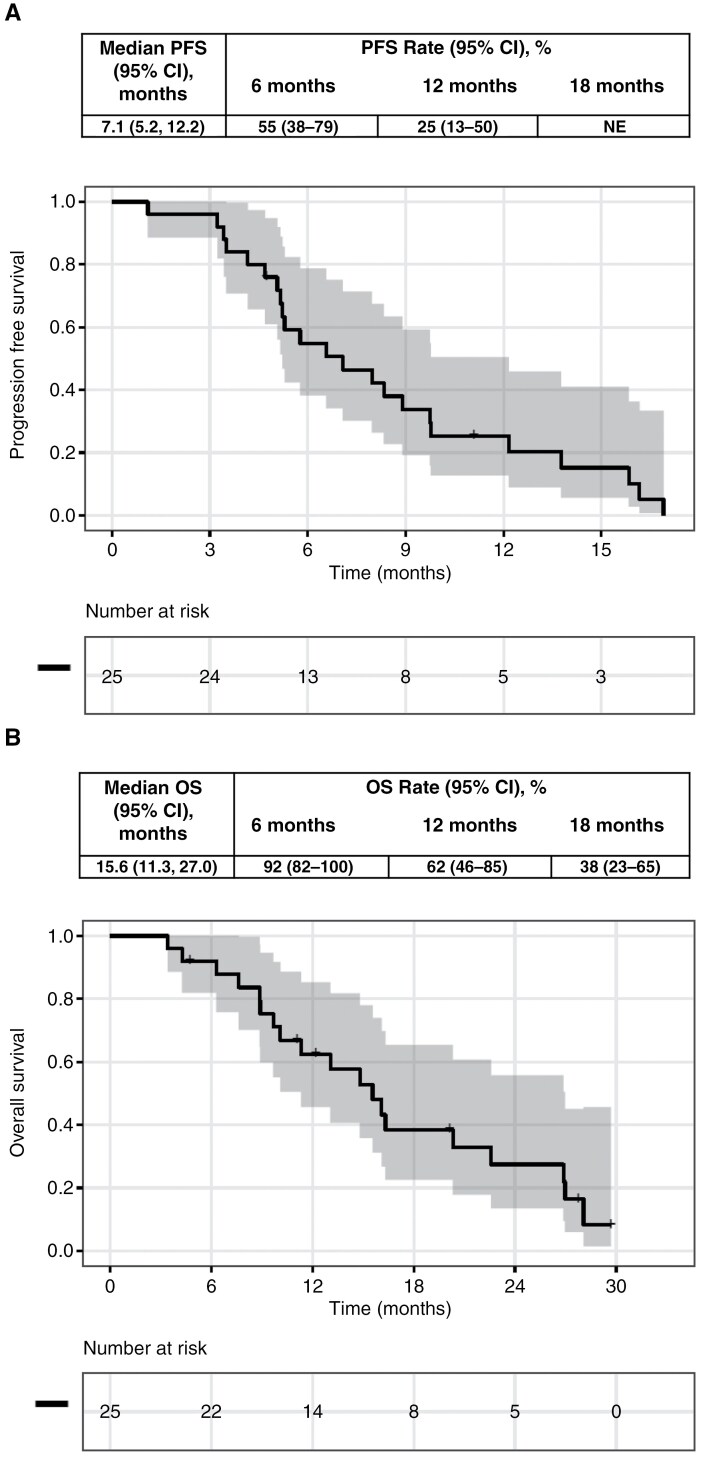
**PFS and OS in all patients. A,** The median PFS, PFS rates at 6, 12, and 18 months, and the Kaplan–Meier curve for PFS. **B,** The median OS, OS rates at 6, 12, and 18 months, and the Kaplan–Meier curve for OS.

Using RANO to assess response, 1 patient (4%) had a complete response and 15 (60%) had a partial response based on findings of post-treatment brain MRI, yielding a rate of objective response of 64% (95% CI, 43–81). Disease control rates, defined by complete response + partial response + stable disease, were 96% (95% CI, 78–100) (Table 3). The median duration of response (range) was 5.57 (0–17) months. With a median follow-up of 6.6 months (range 1.1–16.9) for PFS and 13.1 months (range 3.4–32.9) for OS, median PFS and OS were 7.1 months (95% CI, 5.2–12.2) and 15.6 months (95% CI, 11.3–27.0), respectively (Figure 2). The 6 months and 12 months PFS were 55% (95% CI, 38–79%) and 25% (95% CI, 13–50%). Six months OS was 92% (95% CI, 82–100%), 9 months OS was 75% (95% CI, 60–95%), 12 months OS was 62% (95% CI, 46–85%), and 18 months OS was 38% (95% CI: 26–65%).

**Table 3.** Best Overall Response and Disease Control Rate

Response	Patients (*N* = 25)
Best Overall Response, *n* (%)	
Complete response	1 (4)
Partial response	15 (60)
Stable disease	8 (32)
≥ 12 weeks	2 (8)
≥ 24 weeks	2 (8)
Progressive disease	1 (4)
Disease control rate	
Disease control rate, *n* (%)	24 (96)
95% CI	78–100

### Tumor Biospecimen Analysis

TMB and microsatellite status data were available in 14 patients. Microsatellite instability was not detected in any of the tested tumor specimens. High TMB (≥ 10 mutations/megabase [mt/Mb]) was observed in one patient. This low frequency prohibited any meaningful assessment of treatment efficacy by TMB in this study. Analysis of this patient’s tumor from initial diagnosis was consistent with an *IDH* wildtype, *MGMT* promoter methylated HGG, and TMB of 18 mt/Mb. This patient received 30 cycles of study treatment which resulted in stable disease and clinical improvement. The subject experienced disease progression 484 days after the start of clinical trial treatment in this study and passed away 136 days after disease progression.

### Recurrence Pattern

Imaging data from 21 patients treated at Moffit Cancer Center were used for this exploratory analysis; imaging data of 19 patients were evaluable. All of the patients received prior radiotherapy with a median dose of 6000 cGy in 30 fractions to the region receiving HFSRT in this study. The median GTV was 6.11 cm^3^ (range, 0.10–35.4 cm^3^). The median PTV which received 3000 cGy in 5 fractions was 12.50 cm^3^ (range, 6.87–123.53 cm^3^). The radiographic median follow-up was 13.1 months (range, 1.05–49.70 months). Nine (47.4%) and 15 patients (78.9%) had progression of disease on imaging at 6 months and 12 months, respectively. All of the patients developed infield/marginal progression of disease defined as new enhancement within or overlapping with the region of 80% isodose line. Among these, 17 patients (89.5%) had infield/marginal progression of disease, while 2 patients (10.5%) had both infield/marginal and distant intracranial progression of disease on imaging.

## Discussion

GBM and other HGGs are shown to be profoundly resistant to immunotherapeutics such as vaccines and immune checkpoint inhibitors. Hypofractionated stereotactic radiation therapy combined with immunotherapy has been postulated as a potential strategy to enhance in vivo immune responses against tumor cells. Furthermore, re-irradiation in the form of fractionated stereotactic radiation therapy has been one of the treatment options for patients with recurrent GBM who have very limited systemic therapies avialable.^25,26^ While the potential harm to normal brain tissue previously exposed to high-dose radiation is a concern in this setting, HFSRT has been proven to be a safe salvage option alone and/or in combination with bevacizumab or immunotherapy.^22,25,27^ In support of this concept, our group previously reported that the combination of HFSRT (600 cGy × 5 fractions) with pembrolizumab and bevacizumab is feasible and warrants further investigation in exploring optimal radiation dose/fractionation and timing combined with immunotherapeutic agents.^9^ Our current study further confirms the safety and feasibility of combining HFSRT with dual immune checkpoint inhibitors and bevacizumab. Overall, this combination regimen was surprisingly well tolerated. With respect to safety, the primary endpoint, the combination of ipilimumab and nivolumab at the same dose and frequency adopted in this clinical trial has shown to be associated with immune-mediated TRAEs of grade ≥ 3 in 34% of patients with melanoma.^28^ Ipilimumab 1 mg/kg and nivolumab 3 mg/kg were also studied in a small exploratory phase I cohort of CheckMate 143 clinical trial enrolling patients with first recurrence of GBM where the incidence of immune-mediated TRAEs of grade ≥ 3 was 30%.^29^ In our study, TRAEs of grade 3 or 4 were observed in 48%, however, the most common TRAE grade ≥ 3 was hypertension, a known adverse event of bevacizumab, and this adverse effect was adequately controlled with anti-hypertensive medications with no need for treatment interruption or discontinuation for the affected patients. There was no increase in the incidence of immune-mediated TRAE grade ≥ 3. Notably, despite re-irradiation with HFSRT combining with dual immune checkpoint inhibitors, we did not observe delayed radiation treatment effect or radiation necrosis.

Although efficacy was not the primary endpoint, our study demonstrated signals of improved response compared to the results of published clinical trials investigating single-agent anti-PD-1/PD-L1 or combinations of anti-PD-1/PD-L1 antibodies with bevacizumab where the median OS of approximately 9 months was no better than the standard of care. In our study, the median OS was 15.6 months with a 12-month OS rate of 62%. It is possible that the improved efficacy in this study may be due to the highly selected patient population in a phase 1 trial setting, small numbers of patients, or small gross tumor volume (< 4cm^3^). In addition, almost half of enrolled patients had undergone surgical resection prior to enrollment. Nevertheless, given the positive results of our two consecutive trials that treated a total number of 58 patients with similar combination regimen, we hypothesize potential therapeutic efficacy signals exist when combining immunotherapy with hypofractionated radiation, and this approach warrants further investigation.

Published literature showed the immunomodulation effect of hypofractionated radiotherapy.^30^

Preclinical data supporting the combination of hypofractionated radiation with immunotherapy have suggested that moderate ablative radiation doses such as 700 to 800 cGy per fraction lead to lower regulatory T cells (Tregs), better tumor control, and may enhance abscopal effects.^14,31^ In our study, all patients received 3000 cGy (600 cGy × 5 daily fractions) to the Planning Tumor Volume (T1post contrast-enhancing tissue + re-resection cavity + margin) based on available safety data from combining HFSRT and bevacizumab.^22^ Our study also allowed gross tumor up to 0.5 cm^3^ receiving 4000 cGy (800 cGy in 5 daily fractions) as a boost as a strategy to enhance immune response. To achieve this, gross tumor volume (GTV) received a higher dose as expected. Dosimetric evaluation showed that the average dose to the periphery of GTV was 3500 cGy suggesting that it is safe to deliver hypofractionated ablative doses higher than 600 cGy in 5 fractions to the gross tumor in the reirradiation setting. Analysis of patterns of recurrence from our study showed that locoregional control continues to be a challenge, with all of the patients developing infield/marginal progression despite high-dose hypofractionated radiation therapy. Selection of appropriate dose-fractionation is limited by the nature of re-irradiation and the risk of normal brain tissue injury. In this trial, we chose a moderate hypofractionated radiotherapy regimen that has been reported to be feasible and safe and has been investigated in preclinical studies and in combination with anti-CTLA-4 blockade.^13,22^ Alternative hypofractionated radiotherapy regimens such as 8 Gy x 3 fractions to the regions of enhancing abnormality which may lead to improved immunogenicity may be feasible. Future studies may also attempt to investigate further ultra-hypofractionated dose schema and/or expansion of treatment volumes to encompass regions of pathologic T2/FLAIR abnormalities that may harbor microscopic extension to improve coverage of recurrent disease using techniques such as simultaneously integrated boost. Novel dose fraction regimens may further enhance response synergistically. Our group has explored novel radiotherapeutic approaches and recently published a hypothetical HFSRT regimen driven by mathematical modeling based on clinical data from our HFSRT with pembrolizumab and bevacizumab clinical trial and proposed that intermittently delivered fractions may be an innovative approach to further optimize response to therapy.^32^ Thus, investigation in either further dose-escalated hypofractionated (stereotactic) regimens or altered fractionation schema may be needed when combined with immunotherapy to improve locoregional control or to enhance survival in patients with recurrent HGG.

There are several limitations in our study. First, while we proposed that combining high-dose HFSRT with dual immunotherapy (ipilimumab and nivolumab) and a VEGF inhibitor (bevacizumab) may enhance immunological responses on the basis of preclinical models in gliomas, we lacked sufficient tumoral tissue or serum biomarkers during treatment to sufficiently answer this question in our trial. Other limitations in our study are related to its phase I nature, including small sample size and highly selected patient population, making generalizability of the result challenging. The small number of patients and the correspondingly large confidence intervals of trial endpoints from the analysis make definitive conclusions regarding efficacy premature. Moreover, almost half of enrolled patients had surgical resection prior to clinical trial enrollment resulting in a smaller volume of recurrent tumor which may impact response to study treatment.^33^ Finally, the patient population is heterogeneous and included patients who have been heavily pretreated or have a tumor harboring *IDH* mutation (2 subjects), which in conjunction with inter-tumor and intra-tumor heterogeneities may have a significant difference in baseline prognosis.

In summary, this trial evaluated the safety and efficacy of hypofractionated stereotactic re-irradiation in combination with ipilimumab, nivolumab, and bevacizumab in bevacizumab-naïve recurrent high-grade glioma patients. This combination demonstrated promising therapeutic signal and resulted in improved clinical benefit for these patients. While our trial demonstrated encouraging improvements in median OS, these results will require additional validation in a randomized clinical trial. This study, combined with our previously published trial and other published data, underscores the potential benefit of utilizing stereotactic radiosurgery in optimizing the efficacy of immunotherapy in patients with high-grade gliomas.^9^

## Data Availability

Data sets and other files generated, analyzed, or used during this study are available from the corresponding author upon reasonable request.
